# Diagnostic delays and suicidal thoughts and behaviors among young adults with a history of mood disorders

**DOI:** 10.3389/fpsyt.2026.1894148

**Published:** 2026-07-22

**Authors:** David R. Pletta, Sari L. Reisner, Caitlynn Feng, Cassandra Morrow, Maya C. Clark, Badar Omar, Alex S. Keuroghlian, Rena Xu

**Affiliations:** 1 Department of Epidemiology, University of Michigan School of Public Health, Ann Arbor, MI, United States; 2 Center for Social Epidemiology and Population Health, University of Michigan, Ann Arbor, MI, United States; 3 Eisenberg Family Depression Center, University of Michigan, Ann Arbor, MI, United States; 4 Department of Urology, Boston Children’s Hospital, Harvard Medical School, Boston, MA, United States; 5 Department of Psychiatry, MaineHealth, Portland, ME, United States

**Keywords:** diagnostic delay, mood disorders, non-suicidal self-injury, suicidal ideation, suicide attempt, young adults

## Abstract

**Introduction:**

Suicidal thoughts and behaviors (STBs) are prevalent among young adults with mood disorders. This study examined whether length of diagnostic delay was independently associated with risk for STBs.

**Materials and methods:**

Data were from a national online survey of US adults ages 18–35 years with a lifetime mood disorder diagnosis (N = 464). Modified Poisson regression estimated adjusted risk ratios (aRRs) for associations between diagnostic delay (<1, 1-2, 3-4, 5+ years) and three lifetime and past 6-month STB outcomes: suicidal ideation (SI), suicide attempt (SA), and non-suicidal self-injury (NSSI).

**Results:**

Median diagnostic delay was 3 years (IQR = 0–7 years). Delays of 1–2 years (aRR=1.23, 95% CI = 1.03-1.47, p=0.021) and 3–4 years (aRR=1.26, 95% CI = 1.07-1.49, p=0.006) were associated with higher lifetime SI risk. A 3-4-year delay was associated with past 6-month SI (aRR=1.37, 95% CI = 1.02-1.85, p=0.038). Diagnostic delay was not significantly associated with SA or NSSI. Sexual minority status and adverse childhood experiences were associated with elevated risk for multiple STB outcomes, and social support was protective.

**Discussion:**

Diagnostic delay may be associated with elevated risk for SI in young adults with mood disorders; longitudinal studies are needed to further investigate the temporal relationship between diagnostic delays and risk for STBs within this population.

## 1 Introduction

Suicidal thoughts and behaviors (STBs), including suicidal ideation (SI), suicide attempts (SA), and non-suicidal self-injury (NSSI), are a significant and growing public health concern among young adults in the United States (US). Suicide is the second leading cause of death among individuals ages 10–34 years (1). Rates of SI, SA, and NSSI have increased substantially over the past two decades. Data from the National Survey on Drug Use and Health (NSDUH) showed that the prevalence of past-year SI among adults ages 18–25 years increased by approximately 47% (8.3% to 12.2%) between 2015 and 2019 (2), reaching 12.6% in 2024 (3). Despite this burden, only 40.6% of young adults ages 18–25 years with SI reported receiving mental healthcare during this period (2). Between 2008 and 2019, the adjusted odds of SA increased 81% among young adults ages 18–25 years, with 35%-46% of adults with an SA reporting an unmet need for mental health services (4). In 2023, an estimated 2.2 million adults reported a SA, with the highest prevalence among those ages 18–25 years (2.0%) (3). NSSI, which is most prevalent among adolescents and young adults ages 14–24 years (5), has been found to be a strong predictor of progression from SI to SA (6).

Certain subpopulations face elevated risk for STBs. Gender minority (transgender, nonbinary, and gender diverse; TGD) young adults report SI rates as high as 66% (7), and sexual minority individuals (lesbian, gay, bisexual, queer, and other non-heterosexual individuals) have significantly greater odds of all forms of suicidal behaviors compared to heterosexual peers (8). Prior research has also identified multiple psychosocial and clinical risk factors for STBs among U.S. adults, including unemployment, serious psychological distress (based on a clinically elevated score on the 6-item Kessler Psychological Distress Scale) (9), major depressive episodes (10), adverse childhood experiences (11, 12), and comorbid physical and psychiatric health conditions (4). In contrast, studies have found that perceived social support is protective against STBs in young adults (13, 14).

Depressive disorders are strongly associated with risk for STBs (15). The prevalence of major depressive episodes among adults ages 18–25 years has nearly doubled since the mid-2000s, increasing from 8.8% to 15.9% in 2024 (3, 16), with estimates as high as 17.2% in 2020 prior to NSDUH methodology changes (17). However, increases in depression prevalence have not been matched by improvements in treatment engagement, and a substantial and widening treatment gap persists (17). From 2015 to 2019, depression prevalence increased significantly among young adults without commensurate increases in help-seeking (17). Approximately half of young adults ages 18–25 with any mental illness received no treatment in 2024, with access disparities further stratified by race/ethnicity and sexual and gender minority status (3). This treatment gap is clinically consequential, as a shorter duration of untreated depression is associated with significantly higher odds of treatment response and remission (18), potentially mitigating prolonged risk for STBs.

Despite the central role of mood disorders in STB risk and the potential impact of earlier diagnosis and treatment for STB risk reduction for young adults living with mood disorders, research on diagnostic delays has largely focused on specific disorders, such as bipolar disorder (BD) (19) and first-episode psychosis (20), rather than capturing possible heterogeneity within mood disorders as a broader class of psychiatric conditions (i.e., inclusive of those with major depressive disorder [MDD], persistent depressive disorder [PDD], and BD). As a result, the extent to which diagnostic delay for a mood disorder is independently associated with SI, SA, and NSSI remains underexplored. Understanding whether diagnostic delay is associated with risk for suicidal thoughts and behaviors could inform strategies for the timely identification and treatment of these conditions in young adults.

The aims of the current study were twofold: first, to examine sociodemographic, clinical, and psychosocial risk factors for SI, SA, and NSSI among a national online convenience sample of US young adults with a lifetime mood disorder diagnosis; and second, to assess whether diagnostic delay, defined as the time from first depressive symptoms to first diagnosis, was independently associated with these outcomes after adjusting for established risk factors.

## 2 Materials and methods

### 2.1 Study design

This cross-sectional study used data from a national online survey of US adults ages 18–35 years conducted via the online research platform Prolific in May 2025. A random sample of eligible participants was invited to complete a self-administered electronic survey assessing sociodemographic characteristics, mood disorder history and treatment experiences, suicidal thoughts and behaviors, and psychosocial factors. The study was approved by the Institutional Review Board of Boston Children's Hospital (IRB-P00049962). All respondents provided electronic informed consent and were compensated US $3 for their participation. This study followed the Strengthening the Reporting of Observational Studies in Epidemiology (STROBE) guidelines for reporting observational research (21).

### 2.2 Participants

Eligible participants were US adults ages 18–35 years who were able to provide informed consent and complete the survey in English. The age range of 18–35 years was selected to encompass both emerging and early adulthood. A total of 959 respondents met the eligibility criteria and completed the survey. Of these participants, 464 reported a lifetime diagnosis of a mood disorder by a mental health provider, including MDD, PDD, BD type I, II, or type unknown, or a mood disorder not otherwise specified. Participants also reported their age at first symptom emergence (i.e., symptom onset) and age at first diagnosis. The current study was restricted to this subsample (n=464) to examine associations between diagnostic delay and suicidal thoughts and behaviors.

### 2.3 Measures

#### 2.3.1 Sociodemographic, clinical, and psychosocial factors

Age was collected as a continuous integer and categorized as 18-22, 23-25, 26-29, and 30–35 years (reference). Race/ethnicity included Black/African American, Hispanic/Latino, multiracial, or another racial/ethnic identity, and dichotomized as White (reference) versus person of color due to sparse cell sizes in outcome-stratified analyses. Gender identity was categorized as cisgender woman (reference), cisgender man, transmasculine, transfeminine, and nonbinary. Sexual minority status was dichotomized as heterosexual (reference) versus LGBQ+ (lesbian, gay, bisexual, queer, or another non-heterosexual identity). Additional sociodemographic variables included educational attainment (less than a bachelor’s degree [reference] vs. bachelor’s degree or higher), employment status (employed or other [reference] vs. unemployed), US Census region (Midwest, Northeast, South [reference], West), and urbanicity (urban [reference], suburban, rural).

Self-rated general health was assessed using a single item from the 12-Item Short Form Health Survey (SF-12) (22) and dichotomized as excellent/very good/good (reference) versus fair/poor. Mental health comorbidities were assessed using a checklist of 10 common non-depressive psychiatric diagnoses (e.g., attention deficit hyperactivity disorder [ADHD], borderline personality disorder, schizophrenia, substance use disorder), which were coded as 0, 1, or 2+ conditions for descriptive analysis and dichotomized as none (reference) versus any in regression models. Adverse childhood experiences (ACEs) were measured using four items adapted from a validated ACEs questionnaire (11), coded as 0, 1, or 2+ for descriptive purposes and dichotomized into none (reference) versus any for modeling.

Perceived social support was assessed using an 8-item subset of the Multidimensional Scale of Perceived Social Support (MSPSS), capturing perceived support from family, friends, and significant others (23). An example item is “There is a special person who is around when I am in need.” Items were rated on a 7-point Likert scale, ranging from very strongly disagree (1) to very strongly agree (7). The scale demonstrated high internal consistency in the sample (standardized Cronbach’s α=0.89). Participants who completed at least 6 of 8 items had their scores included, and summed scores were standardized as z-scores for modeling.

#### 2.3.2 Exposure: diagnostic delay for a mood disorder

Diagnostic delay was defined as the self-reported duration in years from depressive symptom onset to the age at which a participant was first diagnosed with a mood disorder. Participants selected the categorical response option which best described the length of delay they encountered from first experiencing depressive symptoms to first being diagnosed with a mood disorder by a mental health professional: less than 1 year (reference), 1–2 years, 3–4 years, or 5+ years.

Because mood disorders share overlapping depressive symptomatology and care pathways, and because diagnostic delay was defined relative to depressive symptom onset, participants with major depressive disorder, persistent depressive disorder, and bipolar spectrum disorders were analyzed together; the robustness of our models within strata of participants with specific mood disorders was explored in subsequent sensitivity analyses along with our operationalization of diagnostic delay.

#### 2.3.3 Outcomes: suicidal thoughts and behaviors

Six binary outcomes were examined to capture lifetime and past 6-month occurrence of SI, SA, and NSSI. SI was assessed using two items from the Columbia-Suicide Severity Rating Scale (C-SSRS): “Have you wished you were dead or wished you could go to sleep and not wake up?” and “Have you actually had any thoughts of killing yourself?” Participants endorsing either item were classified as having experienced SI. Lifetime endorsement prompted assessment of SI in the past 6 months (24). SA was assessed using an adapted item from the Study to Assess Risk and Resilience in Service Members (STARRS) instrument: “Have you ever made a suicide attempt (purposefully hurt yourself with at least some intention to die)?” with an affirmative lifetime response prompting a past 6-month assessment (25). NSSI was measured using an adapted STARRS item: “Did you ever do something to hurt yourself on purpose, but without wanting to die (e.g., cutting yourself, hitting yourself, or burning yourself)?” Lifetime endorsement similarly prompted a past 6-month assessment (25).

### 2.4 Statistical analysis

Descriptive statistics were generated for the full analytic sample. Modified Poisson regression with robust standard errors was used to estimate risk ratios (RRs) and adjusted risk ratios (aRRs), with 95% confidence intervals (CIs) and p-values for each outcome. This approach was selected because logistic regression can overestimate the odds ratio as an approximation of the risk ratio when outcomes are common (prevalence >10%) (26), whereas modified Poisson regression provides consistent estimates of risk ratios regardless of outcome prevalence (27).

Separate regression models were fit for each of the 6 outcomes to examine associations between diagnostic delay and lifetime and past 6-month risk of SI, SA, and NSSI, while controlling for sociodemographic, clinical, and psychosocial covariates. For each outcome, bivariate models were estimated for each predictor individually, followed by a single multivariable model including all predictors simultaneously. Given minimal missingness (<2% for any variable), multivariable models were restricted to complete cases. Multicollinearity was assessed using generalized variance inflation factors (GVIF), with values below 2.5 indicating acceptable levels (28). All analyses were conducted in R (version 4.5.1) with a two-sided statistical significance level of α = 0.05.

Several sensitivity analyses were also conducted to test the robustness of our initial model fitting and findings. To assess the potential impact of improperly homogenizing participants with heterogeneous diagnostic histories, diagnostic-delay models were refit within strata of participants with specific mood disorders (unipolar/no bipolar, bipolar spectrum, major depressive disorder only, and excluding mood disorder not otherwise specified/other). To evaluate the shape of the association, diagnostic delay was additionally modeled as a continuous variable (risk ratio per 1-year increase), compared with a categorical specification to test for departure from linearity, and modeled with restricted cubic splines (knots at 0, 3, and 7 years) (29); models were also refit with the 5+ year category for diagnostic delay subdivided into 5–9 and 10+ years. Given the small number of past 6-month suicide attempt events, a parsimonious model retaining only covariates associated with the outcome at p<0.05 was also fit. Results from these sensitivity analyses are presented in the accompanying **Supplementary Material**.

## 3 Results

### 3.1 Descriptive statistics

#### 3.1.1 Sample characteristics

Descriptive statistics for the analytic sample are presented in **Table 1**. Among participants (N = 464), the largest age group was 30–35 years (42.9%), followed by 26–29 years (30.0%), 23–25 years (18.3%), and 18–22 years (8.8%). The sample was 63.1% White, 18.8% Black/African American, 7.1% Hispanic/Latino, 5.4% multiracial, and 4.7% another racial identity. By gender identity, 50.2% identified as cisgender women, 32.8% as cisgender men, 8.8% as nonbinary, 3.4% as transfeminine, and 3.2% as transmasculine. Nearly half (46.8%) identified as a sexual minority (LGBQ+). Most participants (59.1%) had a bachelor’s degree or higher, and 12.5% were unemployed. By US Census region, 42.7% resided in the South, 22.6% in the West, 18.8% in the Northeast, and 15.9% in the Midwest; 45.9% described their community as suburban, 41.2% as urban, and 12.3% as rural.

**Table 1:** Characteristics of the US nationwide sample of young adults with a history of mood disorders (N = 464).

Characteristic	N=464 (%)
Age group (years)	
18-22	41 (8.8)
23-25	85 (18.3)
26-29	139 (30.0)
30-35	199 (42.9)
Race/ethnicity	
White	293 (63.1)
Black/African American	87 (18.8)
Hispanic/Latino	33 (7.1)
Multiracial	25 (5.4)
Other	22 (4.7)
Missing	4 (0.9)
Gender identity	
Cisgender woman	233 (50.2)
Cisgender man	152 (32.8)
Transmasculine	15 (3.2)
Transfeminine	16 (3.4)
Nonbinary	41 (8.8)
Missing	7 (1.5)
Sexual minority status	
Heterosexual	246 (53.0)
LGBQ+	217 (46.8)
Missing	1 (0.2)
College degree	
< Bachelor’s	168 (36.2)
≥ Bachelor’s	274 (59.1)
Missing	22 (4.7)
Employment status	
Employed/other	395 (85.1)
Unemployed	58 (12.5)
Missing	11 (2.4)
US region	
South	198 (42.7)
West	105 (22.6)
Northeast	87 (18.8)
Midwest	74 (15.9)
Urbanicity	
Urban	191 (41.2)
Suburban	213 (45.9)
Rural	57 (12.3)
Missing	3 (0.6)
Self-rated health (fair/poor)	
Excellent/Very good/Good	321 (69.2)
Fair/Poor	143 (30.8)
Mood disorder type	
Major Depressive Disorder	320 (69.0)
Persistent Depressive Disorder	77 (16.6)
Bipolar Disorder (unsure of type)	48 (10.3)
Bipolar Disorder Type I	25 (5.4)
Bipolar Disorder Type II	25 (5.4)
Mood Disorder NOS	72 (15.5)
Other	32 (6.9)
Mental health comorbidities	
0 conditions	183 (39.4)
1 condition	96 (20.7)
2+ conditions	185 (39.9)
Adverse childhood experiences (ACEs)	
0 ACEs	135 (29.1)
1 ACE	117 (25.2)
2+ ACEs	212 (45.7)
Perceived social support (MSPSS sum), mean (SD)	29.80 (7.73)
Diagnostic delay (symptoms to diagnosis)	
< 1 year	125 (26.9)
1–2 years	99 (21.3)
3–4 years	77 (16.6)
5+ years	163 (35.1)
Suicidal thoughts and behaviors	
Lifetime SI	360 (77.6)
Past 6-month SI	242 (52.2)
Lifetime SA	174 (37.5)
Past 6-month SA	32 (6.9)
Lifetime NSSI	282 (60.8)
Past 6-month NSSI	113 (24.4)

LGBQ+, lesbian, gay, bisexual, queer, or other sexual minority identity; NOS, not otherwise specified; MSPSS, multidimensional scale of perceived social support; SI, suicidal ideation; SA, suicide attempt; NSSI, non-suicidal self-injury. Participants’ MSPSS scaled scores were included if they answered at least 6 of the 8 items included in the adapted instrument.

Nearly one third (30.8%) of participants rated their general health as fair or poor. The most reported mood disorder was major depressive disorder (69.0%), followed by persistent depressive disorder (16.6%) and bipolar disorder of unspecified or unknown type (10.3%). Non-depressive mental health comorbidities were common: 20.7% reported one comorbid condition and 39.9% reported two or more. Over two-thirds (70.9%) reported at least one ACE (25.2% one, 45.7% two or more). The mean perceived social support score was 29.8 (SD = 7.7; possible range 8-56).

#### 3.1.2 Diagnostic delay and prevalence of suicidal thoughts and behaviors

Diagnostic delay varied substantially within the sample (N = 464): 26.9% reported a delay of less than 1 year from first depressive symptoms to first diagnosis, 21.3% reported 1–2 years, 16.6% reported 3–4 years, and 35.1% reported delays of 5 or more years. The median diagnostic delay was 3 years (interquartile range [IQR]=0–7 years). Suicidal thoughts and behaviors were highly prevalent. Overall, 360 (77.6%) of participants endorsed lifetime SI, 174 (37.5%) reported lifetime SA, and 282 (60.8%) endorsed lifetime NSSI. Among those with a lifetime history, 67.2% reported past 6-month SI (n=242/360), 18.4% reported past 6-month SA (n=32/174), and 40.1% reported past 6-month NSSI (n=113/282).

#### 3.1.3 Diagnostic delay and suicidal thoughts and behaviors

We examined whether diagnostic delay duration was independently associated with SI, SA, and NSSI after adjustment for sociodemographic, clinical, and psychosocial covariates. Estimates for the association between these outcomes and length of diagnostic delay were generated using a reference group of participants who reported <1 year of delay between first experiencing depressive symptoms and first being diagnosed with a mood disorder.

### 3.2 Model results

#### 3.2.1 Suicidal ideation

Bivariate and multivariable results for lifetime and past 6-month SI are presented in **Table 2**. In our adjusted model for lifetime SI, participants with diagnostic delays of 1–2 years (aRR=1.23, 95% CI = 1.03-1.47, p=0.021) and 3–4 years (aRR=1.26, 95% CI = 1.07-1.49, p=0.006) had significantly higher risk for lifetime SI relative to those who experienced a diagnostic delay <1 year. A delay of 5+ years did not reach statistical significance (aRR=1.06, 95% CI = 0.90-1.25, p=0.478). Sexual minority status (aRR=1.14, 95% CI = 1.02-1.28, p=0.024) and any ACEs (aRR=1.17, 95% CI = 1.00-1.36, p=0.045) were associated with significantly elevated risk for lifetime SI, while identifying as person of color (aRR=0.84, 95% CI = 0.74-0.96, p=0.007) and greater perceived social support (aRR=0.89, 95% CI = 0.85-0.94, p<0.001) demonstrated a protective association.

**Table 2:** Bivariate and multivariable modified Poisson regression model results for the association between length of diagnostic delay and risk for lifetime and past 6-month suicidal ideation (SI) among young adults with a history of mood disorders.

Predictor	Lifetime SI (n=413)		Past 6-month SI (n=409)	
	Bivariate RR (95% CI), p-value	Multivariable aRR (95% CI), p-value	Bivariate RR (95% CI), p-value	Multivariable aRR (95% CI), p-value
Age group (years)				
18-22	0.82 (0.65-1.04), p=0.098	0.81 (0.61-1.07), p=0.135	0.64 (0.41-1.01), p=0.058	0.64 (0.37-1.13), p=0.122
23-25	0.94 (0.81-1.08), p=0.362	0.93 (0.80-1.08), p=0.345	0.96 (0.75-1.24), p=0.778	0.97 (0.74-1.27), p=0.814
26-29	0.98 (0.87-1.09), p=0.661	0.99 (0.88-1.11), p=0.856	1.10 (0.90-1.33), p=0.344	1.17 (0.95-1.45), p=0.143
30-35	Reference	Reference	Reference	Reference
Race/ethnicity				
Person of Color	**0.86 (0.77-0.97), p=0.010**	**0.84 (0.74-0.96), p=0.007**	**0.76 (0.62-0.93), p=0.007**	**0.76 (0.61-0.94), p=0.014**
White	Reference	Reference	Reference	Reference
Gender identity				
Cisgender man	**0.88 (0.78-1.00), p=0.047**	0.91 (0.79-1.04), p=0.147	0.84 (0.68-1.05), p=0.124	0.88 (0.70-1.10), p=0.260
Transmasculine	1.10 (0.88-1.38), p=0.387	1.07 (0.79-1.45), p=0.653	1.27 (0.85-1.90), p=0.248	1.26 (0.73-2.20), p=0.406
Transfeminine	1.11 (0.90-1.37), p=0.310	1.04 (0.80-1.36), p=0.753	**1.43 (1.03-1.98), p=0.033**	1.32 (0.83-2.09), p=0.247
Nonbinary	**1.21 (1.10-1.34), p<0.001**	1.04 (0.90-1.20), p=0.618	1.25 (0.97-1.62), p=0.086	0.88 (0.64-1.20), p=0.420
Cisgender woman	Reference	Reference	Reference	Reference
Sexual minority status				
LGBQ+	**1.21 (1.10-1.34), p<0.001**	**1.14 (1.02-1.28), p=0.024**	**1.37 (1.15-1.64), p<0.001**	1.17 (0.96-1.44), p=0.124
Heterosexual	Reference	Reference	Reference	Reference
College degree				
≥ Bachelor’s	0.98 (0.88-1.08), p=0.636	1.05 (0.94-1.18), p=0.374	0.87 (0.73-1.05), p=0.142	1.03 (0.84-1.26), p=0.761
< Bachelor’s	Reference	Reference	Reference	Reference
Employment status				
Unemployed	1.09 (0.95-1.24), p=0.219	1.03 (0.90-1.17), p=0.711	**1.33 (1.08-1.64), p=0.006**	1.23 (0.99-1.52), p=0.063
Employed/other	Reference	Reference	Reference	Reference
US region				
West	0.99 (0.87-1.12), p=0.823	1.01 (0.87-1.16), p=0.941	0.93 (0.73-1.18), p=0.542	1.05 (0.81-1.35), p=0.713
Northeast	1.03 (0.90-1.17), p=0.675	1.02 (0.88-1.18), p=0.837	1.13 (0.91-1.41), p=0.270	1.22 (0.94-1.58), p=0.134
Midwest	0.93 (0.80-1.09), p=0.386	0.92 (0.78-1.09), p=0.330	0.92 (0.70-1.21), p=0.554	0.95 (0.72-1.27), p=0.748
South	Reference	Reference	Reference	Reference
Urbanicity				
Suburban	1.02 (0.92-1.14), p=0.646	1.03 (0.91-1.16), p=0.647	1.07 (0.88-1.29), p=0.508	1.19 (0.96-1.47), p=0.119
Rural	0.98 (0.83-1.16), p=0.817	0.86 (0.72-1.04), p=0.118	1.16 (0.89-1.51), p=0.282	1.05 (0.80-1.38), p=0.741
Urban	Reference	Reference	Reference	Reference
Self-rated health (fair/poor)				
Fair/Poor	**1.14 (1.03-1.25), p=0.009**	1.05 (0.95-1.17), p=0.327	**1.25 (1.05-1.48), p=0.013**	1.05 (0.86-1.27), p=0.640
Excellent/Very good/Good	Reference	Reference	Reference	Reference
Mental health comorbidities				
≥ 1 condition	**1.12 (1.01-1.25), p=0.031**	0.97 (0.87-1.10), p=0.668	1.17 (0.97-1.40), p=0.105	0.88 (0.72-1.09), p=0.251
0 conditions	Reference	Reference	Reference	Reference
Adverse childhood experiences (ACEs)				
≥ 1 ACE	**1.27 (1.11-1.45), p<0.001**	**1.17 (1.00-1.36), p=0.045**	**1.45 (1.15-1.83), p=0.002**	1.29 (0.99-1.69), p=0.062
0 ACEs	Reference	Reference	Reference	Reference
Perceived social support (MSPSS z-score)				
Per 1 SD increase	**0.89 (0.86-0.93), p<0.001**	**0.89 (0.85-0.94), p<0.001**	**0.81 (0.75-0.87), p<0.001**	**0.82 (0.74-0.91), p<0.001**
Diagnostic delay (symptoms to diagnosis)				
< 1 year	Reference	Reference	Reference	Reference
1–2 years	**1.26 (1.07-1.49), p=0.005**	**1.23 (1.03-1.47), p=0.021**	**1.39 (1.03-1.86), p=0.029**	1.25 (0.90-1.72), p=0.179
3–4 years	**1.42 (1.22-1.65), p<0.001**	**1.26 (1.07-1.49), p=0.006**	**1.73 (1.31-2.29), p<0.001**	**1.37 (1.02-1.85), p=0.038**
5+ years	**1.25 (1.07-1.45), p=0.005**	1.06 (0.90-1.25), p=0.478	**1.49 (1.14-1.93), p=0.003**	1.13 (0.85-1.51), p=0.391

RR, risk ratio; aRR, adjusted risk ratio; CI, confidence interval; LGBQ+, lesbian, gay, bisexual, queer, or other sexual minority identity. Bolding indicates p<0.05.

In our adjusted model for past 6-month SI, a 3-4-year diagnostic delay was associated with 37% higher risk compared to those with a delay less than 1 year (aRR=1.37, 95% CI = 1.02-1.85, p=0.038). Identifying as a person of color (aRR=0.76, 95% CI = 0.61-0.94, p=0.014) and greater perceived social support (aRR=0.82, 95% CI = 0.74-0.91, p<0.001) remained protective.

#### 3.2.2 Suicide attempt

Results for lifetime and past 6-month SA are shown in **Table 3**. Diagnostic delay was not significantly associated with either lifetime or past 6-month SA. Factors associated with increased risk of lifetime SA included younger age (26–29 vs. 30–35 years; aRR=1.40, 95% CI = 1.04-1.89, p=0.026), sexual minority status (aRR=1.47, 95% CI = 1.12-1.92, p=0.005), and any ACEs (aRR=1.62, 95% CI = 1.09-2.41, p=0.017). Cisgender men had lower risk of lifetime SA relative to cisgender women (aRR=0.70, 95% CI = 0.50-0.97, p=0.034).

**Table 3:** Bivariate and multivariable modified Poisson regression model results for the association between length of diagnostic delay and risk for lifetime and past 6-month suicide attempt (SA) among young adults with a history of mood disorders.

Predictor	Lifetime SA (n=413)		Past 6-month SA (n=413)	
	Bivariate RR (95% CI), p-value	Multivariable aRR (95% CI), p-value	Bivariate RR (95% CI), p-value	Multivariable aRR (95% CI), p-value
Age group (years)				
18-22	1.01 (0.63-1.64), p=0.954	1.14 (0.65-1.98), p=0.644	1.04 (0.31-3.55), p=0.950	2.21 (0.49-9.95), p=0.301
23-25	1.15 (0.83-1.61), p=0.403	1.12 (0.79-1.60), p=0.531	0.67 (0.22-2.00), p=0.471	0.72 (0.20-2.59), p=0.611
26-29	1.28 (0.97-1.69), p=0.076	**1.40 (1.04-1.89), p=0.026**	1.12 (0.52-2.42), p=0.763	1.32 (0.55-3.14), p=0.537
30-35	Reference	Reference	Reference	Reference
Race/ethnicity				
Person of Color	0.86 (0.67-1.11), p=0.254	0.98 (0.75-1.29), p=0.906	0.80 (0.39-1.65), p=0.542	1.08 (0.51-2.31), p=0.839
White	Reference	Reference	Reference	Reference
Gender identity				
Cisgender man	**0.67 (0.49-0.91), p=0.010**	**0.70 (0.50-0.97), p=0.034**	1.06 (0.46-2.43), p=0.888	0.94 (0.35-2.56), p=0.908
Transmasculine	0.99 (0.50-1.96), p=0.980	0.85 (0.39-1.81), p=0.667	2.39 (0.54-10.51), p=0.249	1.25 (0.09-18.14), p=0.870
Transfeminine	**1.86 (1.32-2.61), p<0.001**	1.46 (0.88-2.41), p=0.145	**6.72 (2.85-15.86), p<0.001**	**4.24 (1.17-15.36), p=0.028**
Nonbinary	1.15 (0.79-1.67), p=0.466	0.66 (0.40-1.10), p=0.110	0.87 (0.20-3.85), p=0.859	0.39 (0.05-3.24), p=0.383
Cisgender woman	Reference	Reference	Reference	Reference
Sexual minority status				
LGBQ+	**1.64 (1.29-2.09), p<0.001**	**1.47 (1.12-1.92), p=0.005**	1.28 (0.66-2.52), p=0.465	1.38 (0.64-2.96), p=0.415
Heterosexual	Reference	Reference	Reference	Reference
College degree				
≥ Bachelor’s	0.94 (0.74-1.21), p=0.644	1.01 (0.77-1.32), p=0.955	1.53 (0.69-3.42), p=0.296	1.40 (0.59-3.30), p=0.448
< Bachelor’s	Reference	Reference	Reference	Reference
Employment status				
Unemployed	1.07 (0.76-1.52), p=0.692	1.15 (0.80-1.65), p=0.457	0.70 (0.22-2.28), p=0.559	1.31 (0.36-4.79), p=0.678
Employed/other	Reference	Reference	Reference	Reference
US region				
West	0.82 (0.59-1.14), p=0.242	0.92 (0.65-1.32), p=0.662	1.06 (0.48-2.33), p=0.883	1.30 (0.53-3.23), p=0.569
Northeast	0.99 (0.72-1.36), p=0.961	1.09 (0.78-1.53), p=0.624	0.71 (0.27-1.90), p=0.496	0.95 (0.29-3.20), p=0.940
Midwest	0.96 (0.68-1.35), p=0.818	0.98 (0.67-1.44), p=0.924	0.33 (0.08-1.45), p=0.143	0.25 (0.03-2.26), p=0.217
South	Reference	Reference	Reference	Reference
Urbanicity				
Suburban	0.85 (0.66-1.10), p=0.212	0.80 (0.60-1.07), p=0.134	**0.22 (0.09-0.54), p<0.001**	**0.28 (0.10-0.80), p=0.018**
Rural	1.04 (0.73-1.49), p=0.810	0.81 (0.54-1.24), p=0.335	0.28 (0.07-1.17), p=0.082	0.34 (0.06-1.90), p=0.220
Urban	Reference	Reference	Reference	Reference
Self-rated health (fair/poor)				
Fair/Poor	**1.27 (1.00-1.62), p=0.048**	1.17 (0.90-1.51), p=0.239	1.18 (0.58-2.38), p=0.653	1.03 (0.43-2.49), p=0.947
Excellent/Very good/Good	Reference	Reference	Reference	Reference
Mental health comorbidities				
≥ 1 condition	**1.57 (1.20-2.06), p=0.001**	1.25 (0.92-1.70), p=0.146	0.95 (0.48-1.89), p=0.887	1.07 (0.41-2.74), p=0.896
0 conditions	Reference	Reference	Reference	Reference
Adverse childhood experiences (ACEs)				
≥ 1 ACEs	**2.23 (1.56-3.20), p<0.001**	**1.62 (1.09-2.41), p=0.017**	**6.16 (1.48-25.66), p=0.013**	**5.07 (1.02-25.14), p=0.047**
0 ACEs	Reference	Reference	Reference	Reference
Perceived social support (MSPSS z-score)				
Per 1 SD increase	**0.87 (0.78-0.97), p=0.011**	0.91 (0.80-1.02), p=0.115	0.96 (0.69-1.34), p=0.809	0.90 (0.58-1.39), p=0.621
Diagnostic delay (symptoms to diagnosis)				
< 1 year	Reference	Reference	Reference	Reference
1–2 years	1.04 (0.73-1.47), p=0.833	0.87 (0.60-1.25), p=0.445	1.03 (0.44-2.41), p=0.940	0.90 (0.32-2.54), p=0.844
3–4 years	1.26 (0.90-1.78), p=0.181	0.93 (0.64-1.34), p=0.700	0.74 (0.26-2.07), p=0.563	0.69 (0.18-2.65), p=0.593
5+ years	0.97 (0.71-1.33), p=0.857	0.72 (0.51-1.02), p=0.063	0.49 (0.19-1.23), p=0.128	0.45 (0.13-1.58), p=0.214

RR, risk ratio; aRR, adjusted risk ratio; CI, confidence interval; LGBQ+, lesbian, gay, bisexual, queer, or other sexual minority identity. Bolding indicates p<0.05.

For past 6-month SA, transfeminine gender identity (compared with cisgender women) (aRR=4.24, 95% CI = 1.17-15.36, p=0.028) and any ACEs (aRR=5.07, 95% CI = 1.02-25.14, p=0.047) were associated with increased risk, while suburban residence was protective (aRR=0.28, 95% CI = 0.10-0.80, p=0.018).

#### 3.2.3 Non-suicidal self-injury

Results for lifetime and past 6-month NSSI are presented in **Table 4**. Length of diagnostic delay did not demonstrate a statistically significant relationship with either lifetime or past 6-month NSSI. Factors associated with elevated risk of lifetime NSSI included younger age (23–25 vs. 30–35 years; aRR=1.25, 95% CI = 1.03-1.53, p=0.026), sexual minority status (aRR=1.30, 95% CI = 1.09-1.54, p=0.003), and any ACEs (aRR=1.36, 95% CI = 1.08-1.70, p=0.009). Perceived social support was protective (aRR=0.92, 95% CI = 0.85-0.99, p=0.034).

**Table 4:** Bivariate and multivariable modified Poisson regression model results for the association between length of diagnostic delay and risk for lifetime and past 6-month non-suicidal self-injury (NSSI) among young adults with a history of mood disorders.

Predictor	Lifetime NSSI (n=411)		Past 6-month NSSI (n=410)	
	Bivariate RR (95% CI), p-value	Multivariable aRR (95% CI), p-value	Bivariate RR (95% CI), p-value	Multivariable aRR (95% CI), p-value
Age group (years)				
18-22	0.93 (0.69-1.26), p=0.661	1.00 (0.69-1.43), p=0.987	0.81 (0.38-1.70), p=0.574	0.92 (0.38-2.24), p=0.860
23-25	**1.20 (1.00-1.43), p=0.048**	**1.25 (1.03-1.53), p=0.026**	**1.61 (1.08-2.42), p=0.020**	**1.64 (1.04-2.60), p=0.035**
26-29	0.99 (0.82-1.18), p=0.874	1.04 (0.87-1.26), p=0.656	1.24 (0.84-1.84), p=0.277	1.32 (0.87-2.02), p=0.192
30-35	Reference	Reference	Reference	Reference
Race/ethnicity				
Person of Color	0.90 (0.77-1.06), p=0.213	0.96 (0.81-1.14), p=0.635	0.78 (0.55-1.11), p=0.173	0.77 (0.52-1.14), p=0.197
White	Reference	Reference	Reference	Reference
Gender identity				
Cisgender man	**0.76 (0.63-0.92), p=0.005**	0.88 (0.72-1.08), p=0.237	0.79 (0.53-1.18), p=0.246	0.88 (0.56-1.37), p=0.564
Transmasculine	1.15 (0.82-1.62), p=0.415	0.97 (0.60-1.58), p=0.910	1.10 (0.43-2.77), p=0.848	0.59 (0.14-2.39), p=0.456
Transfeminine	1.26 (0.94-1.68), p=0.120	1.18 (0.79-1.75), p=0.419	**2.19 (1.26-3.82), p=0.006**	**2.12 (1.02-4.40), p=0.043**
Nonbinary	**1.34 (1.13-1.58), p<0.001**	1.03 (0.85-1.25), p=0.767	1.44 (0.88-2.35), p=0.147	0.71 (0.39-1.28), p=0.254
Cisgender woman	Reference	Reference	Reference	Reference
Sexual minority status				
LGBQ+	**1.54 (1.33-1.79), p<0.001**	**1.30 (1.09-1.54), p=0.003**	**2.07 (1.47-2.91), p<0.001**	**1.86 (1.27-2.73), p=0.002**
Heterosexual	Reference	Reference	Reference	Reference
College degree				
≥ Bachelor’s	0.91 (0.79-1.05), p=0.207	1.03 (0.86-1.22), p=0.766	1.12 (0.79-1.58), p=0.526	1.27 (0.83-1.93), p=0.272
< Bachelor’s	Reference	Reference	Reference	Reference
Employment status				
Unemployed	**1.21 (1.01-1.45), p=0.043**	1.03 (0.84-1.27), p=0.778	1.26 (0.81-1.96), p=0.305	1.31 (0.81-2.10), p=0.270
Employed/other	Reference	Reference	Reference	Reference
US region				
West	0.92 (0.75-1.12), p=0.382	1.06 (0.86-1.30), p=0.604	0.92 (0.59-1.42), p=0.696	1.05 (0.65-1.70), p=0.844
Northeast	0.96 (0.78-1.17), p=0.676	1.03 (0.84-1.28), p=0.758	1.21 (0.81-1.83), p=0.352	1.29 (0.81-2.06), p=0.281
Midwest	0.97 (0.78-1.20), p=0.774	0.95 (0.76-1.19), p=0.647	0.89 (0.54-1.47), p=0.651	0.82 (0.47-1.45), p=0.500
South	Reference	Reference	Reference	Reference
Urbanicity				
Suburban	1.13 (0.96-1.33), p=0.127	1.18 (0.99-1.40), p=0.071	**0.70 (0.49-0.98), p=0.039**	0.75 (0.51-1.12), p=0.158
Rural	**1.24 (1.01-1.54), p=0.045**	1.12 (0.87-1.44), p=0.399	0.70 (0.40-1.23), p=0.215	0.56 (0.29-1.07), p=0.080
Urban	Reference	Reference	Reference	Reference
Self-rated health (fair/poor)				
Fair/Poor	**1.17 (1.01-1.35), p=0.039**	1.05 (0.90-1.24), p=0.527	**1.41 (1.02-1.96), p=0.036**	1.20 (0.83-1.73), p=0.327
Excellent/Very good/Good	Reference	Reference	Reference	Reference
Mental health comorbidities				
≥ 1 condition	**1.40 (1.18-1.65), p<0.001**	1.14 (0.94-1.37), p=0.186	1.37 (0.97-1.94), p=0.074	0.92 (0.61-1.38), p=0.692
0 conditions	Reference	Reference	Reference	Reference
Adverse childhood experiences (ACEs)				
≥ 1 ACE	**1.55 (1.26-1.90), p<0.001**	**1.36 (1.08-1.70), p=0.009**	**2.17 (1.36-3.45), p=0.001**	**1.81 (1.05-3.14), p=0.034**
0 ACEs	Reference	Reference	Reference	Reference
Perceived social support (MSPSS z-score)				
Per 1 SD increase	**0.88 (0.82-0.93), p<0.001**	**0.92 (0.85-0.99), p=0.034**	**0.78 (0.68-0.89), p<0.001**	**0.84 (0.70-1.00), p=0.044**
Diagnostic delay (symptoms to diagnosis)				
< 1 year	Reference	Reference	Reference	Reference
1–2 years	1.09 (0.86-1.38), p=0.484	0.96 (0.75-1.22), p=0.722	1.60 (0.96-2.66), p=0.072	1.36 (0.78-2.38), p=0.281
3–4 years	**1.47 (1.19-1.81), p<0.001**	1.21 (0.97-1.52), p=0.087	1.62 (0.94-2.77), p=0.080	1.31 (0.72-2.36), p=0.378
5+ years	1.19 (0.97-1.47), p=0.091	0.99 (0.80-1.24), p=0.956	**1.62 (1.02-2.59), p=0.043**	1.36 (0.81-2.30), p=0.243

RR, risk ratio; aRR, adjusted risk ratio; CI, confidence interval; LGBQ+, lesbian, gay, bisexual, queer, or other sexual minority identity. Bold indicates p<0.05.

Similar patterns were observed for past 6-month NSSI. Younger age (23–25 vs. 30–35 years; aRR=1.64, 95% CI = 1.04-2.60, p=0.035), sexual minority status (aRR=1.86, 95% CI = 1.27-2.73, p=0.002), and any ACEs (aRR=1.81, 95% CI = 1.05-3.14, p=0.034) were associated with increased risk, while perceived social support remained protective (aRR=0.84, 95% CI = 0.70-1.00, p=0.044). Additionally, transfeminine participants had a significantly higher risk of past 6-month NSSI relative to cisgender women (aRR=2.12, 95% CI = 1.02-4.40, p=0.043).

#### 3.2.4 Sensitivity analyses

Sensitivity analyses examined the association between diagnostic delay and SI along three additional lines of inquiry: (1) among subgroups with different diagnostic histories; (2) using alternative operationalizations of diagnostic delay, including directly-reported categories (1-2, 3-4, 5+ years), derived continuous years based on the difference between a participant’s age at first symptoms and age at first diagnosis, and derived granular categories using these continuous years of delay (1-2, 3-4, 5-9, 10+ years); and (3) whether a dose-response relationship could be observed between diagnostic delay and SI when modeled using modified Poisson regression with HC3 robust standard errors and a natural cubic spline with knots at 0, 3, and 7 years of diagnostic delay.

Results suggested we were underpowered to examine our association of interest within strata of participants with specific diagnostic histories (**Supplementary Table 1**). No statistically significant improvement in model fit was observed between using derived continuous years of diagnostic delay relative to our original categorical operationalization (<1, 1-2, 3-4, 5+ years) (**Supplementary Table 2**), and using a more granular categorical coding for diagnostic delay contributed to a loss of power that obscured some of the associations observed in our original models (**Supplementary Table 3**). Finally, no statistically significant dose-response relationship was observed between continuous diagnostic delay and lifetime and past 6-month SI (Lifetime SI omnibus spline Wald *χ*^2^ = 2.88 [df=2], p=0.237; Past 6-month SI omnibus spline Wald *χ*^2^ = 2.82 [df=2], p=0.244) (**Supplementary Figure 1**).

Due to the rare past 6-month suicide attempt outcome (n=32/455; denominator reflects complete-case analytic N), an additional sensitivity analysis was performed with a more parsimonious model, defined as one that retained only covariates that demonstrated a statistically significant bivariate association with the outcome (gender identity, urbanicity, and any ACEs) and diagnostic delay. This parsimonious model yielded consistent null associations for diagnostic delay (**Supplementary Table 4**).

## 4 Discussion

The current study examined whether diagnostic delay for mood disorders was independently associated with SI, SA, and NSSI among a national online convenience sample of US young adults ages 18–35 years. In multivariable models, longer diagnostic delay was associated with a higher risk of both lifetime and recent SI after adjustment for sociodemographic, clinical, and psychosocial factors. In contrast, diagnostic delay was not significantly associated with SA or NSSI. Together, these results suggest that the interval between symptom onset and formal diagnosis may be a marker of elevated suicidal ideation risk, rather than a confirmed causal determinant, in young adults with mood disorders.

The observed association between diagnostic delay and SI is consistent with prior evidence linking longer duration of untreated depression to poorer clinical outcomes, including greater symptom burden and functional impairment (18). In our sample, participants with delays of 1–2 years and 3–4 years had 23% and 26% higher risk of lifetime SI, respectively, than those diagnosed within 1 year, and a delay of 3–4 years was associated with 37% higher risk of past 6-month SI. Prolonged diagnostic delay may extend exposure to untreated depressive symptoms and related cognitive processes such as hopelessness and rumination, which may intensify suicidal thinking (30, 31). The absence of a significant association for delays of 5+ years may reflect heterogeneity within this group, potentially including individuals with less acute symptom trajectories or differing pathways to care, rather than a simple dose-response pattern.

To robustly examine the potential relationship between length of diagnostic delay and risk for SI, we performed sensitivity analyses in which we fit models within strata of participants with specific diagnostic histories, modeled diagnostic delay continuously, compared continuous and categorical specifications, fit restricted cubic splines, and subdivided the 5+ year group into more granular 5–9 and 10+ years groupings (see **Supplementary Material**). These analyses did not support a monotonic dose-response association, associations between per-year diagnostic delay and lifetime and past 6-month SI were statistically non-significant, the categorical specification did not significantly improve fit over the continuous (linear) term, and restricted cubic spline models showed no significant nonlinearity in the relationship between per-year diagnostic delay and lifetime and past 6-month SI, with elevated risk appearing to be the most concentrated around 5 years of delay before subsequently plateauing (**Supplementary Figure 1**).

We did not observe similar associations between diagnostic delay and risk for SA or NSSI. This divergence may be theoretically informative. Although SI often precedes suicidal behavior, the transition from ideation to attempt is shaped by proximal factors such as acute stressors, access to means, and acquired capability emphasized in suicidal ideation-to-action frameworks (31, 32). Similarly, NSSI often serves emotion-regulation functions that may be more closely tied to developmental, interpersonal, and contextual factors than to time-to-diagnosis alone (33). It is also possible that limited statistical power, particularly for recent SA (32 events) and lifetime NSSI at 3–4 years of delay, reduced our ability to detect more modest associations. More broadly, among young people with mood disorders, those who experience suicidal ideation and those who engage in self-harm may represent partially distinct and heterogeneous groups, differing in developmental trajectories and in the functions the behavior serves, which may further explain the divergent associations observed across outcomes.

Consistent with prior literature, sexual minority status and adverse childhood experiences were associated with higher risk across multiple outcomes, while perceived social support was consistently protective (8, 9, 13). Identifying as a person of color was associated with lower risk of both lifetime and recent SI relative to identifying as White. These patterns underscore the importance of examining SI, SA, and NSSI as distinct outcomes rather than combining them into a single suicide risk measure, as their risk profiles and underlying mechanisms may differ. This finding should be interpreted with caution. The “person of color” category aggregates racially and ethnically diverse groups with differing suicide-risk profiles, and this dichotomization, adopted because of small subgroup sizes, may obscure meaningful within-group heterogeneity and be shaped by sampling, measurement, or cultural differences in symptom reporting (34–36).

These findings have important implications for both clinical practice and public health. National data indicate that many young adults with mental health conditions do not receive timely treatment (3). Earlier identification of mood disorders through routine screening in primary care and educational settings, enhanced mental health literacy, and mental healthcare systems that facilitate timely assessment and referral may help reduce diagnostic delays and associated suicidal ideation risk. The protective association of social support further suggests that strengthening peer, family, and community-based support systems may complement formal clinical care approaches and be associated with reduced risk of SI, SA, or NSSI.

Several study limitations warrant consideration. First, the cross-sectional design and lack of survey questions asking about the age at which they experienced SI, SA, or NSSI preclude establishing temporal ordering of exposures and outcomes. Second, all measures were self-reported, including age at symptom onset and diagnosis, introducing potential recall bias. Third, although participants from throughout the US were eligible for the study, we used a convenience sampling strategy, which may limit the generalizability of our findings. Fourth, self-reported psychiatric diagnoses could not be clinically verified, and the measure of diagnostic delay did not capture treatment quality, intensity, continuity, or duration of untreated illness after diagnosis. Relatedly, two self-reported operationalizations of diagnostic delay, a single-item reported interval and a measure derived by differencing recalled ages, agreed only moderately (linear-weighted κ=0.58; **Supplementary Table 3**), underscoring the imprecision of retrospectively dating exact age at symptom onset and diagnosis.

The current study extends the literature by examining diagnostic delay across a range of mood disorders and across distinct suicide-related outcomes in a diverse national online convenience sample of young adults. Future longitudinal studies using validated clinical assessments and more precise timing of diagnosis and treatment are needed to examine whether reducing diagnostic delay lowers subsequent risk of STBs. Overall, these findings suggest that earlier recognition and diagnosis of mood disorders may help identify young adults at elevated risk for SI; however, the results of the current study should be interpreted as non-causal and preliminary, as our use of cross-sectional data hindered our ability to examine how important clinical aspects surrounding access to an accurate mood disorder diagnosis, including the temporal ordering and recency of symptoms, diagnostic delay, and SI, may be associated with longitudinal risk for STBs in young adults with a history of mood disorders.

## Data Availability

The raw data supporting the conclusions of this article will be made available by the authors, without undue reservation.
