# Supplementary material for: Diagnostic delays and suicidal thoughts and behaviors among young adults with a history of mood disorders
Source: Front Psychiatry. 2026 Jul 22;17:1894148. doi: 10.3389/fpsyt.2026.1894148 (PMC13437458; doi:10.3389/fpsyt.2026.1894148)
Supplement: Supplementary file 1 [file DataSheet1.pdf]

Supplementary Material

This Supplementary Material accompanies the manuscript titled, “Diagnostic delays and suicidal thoughts and behaviors among young adults with a history of mood disorders.” All models use modified Poisson regression with HC3 robust standard errors, mirroring the primary analysis. Unless noted, models adjust for the same covariates as the main multivariable models (age, race/ethnicity, gender identity, sexual minority status, college degree, employment, US region, urbanicity, self-rated health, mental health comorbidities, adverse childhood experiences, and perceived social support). Abbreviations: aRR = adjusted risk ratio; CI = confidence interval; SI = suicidal ideation; SA = suicide attempt; NSSI = non-suicidal self-injury.

Supplementary Table S1. Diagnostic-delay associations stratified by mood-disorder subtype.

Mood disorder subset	Outcome	Diagnostic delay	aRR (95% CI)	p-value	N	Events
**Unipolar/no bipolar**	Lifetime SI	1-2 years	1.14 (0.92-1.41)	p=0.224	292	256
		3-4 years	1.13 (0.93-1.38)	p=0.216	292	256
		5+ years	0.96 (0.79-1.17)	p=0.679	292	256
	Past 6-month SI	1-2 years	1.16 (0.77-1.75)	p=0.480	288	171
		3-4 years	1.39 (0.96-2.01)	p=0.085	288	171
		5+ years	1.08 (0.76-1.55)	p=0.665	288	171
	Lifetime SA	1-2 years	0.79 (0.49-1.29)	p=0.345	292	116
		3-4 years	1.00 (0.62-1.63)	p=0.984	292	116
		5+ years	0.70 (0.44-1.10)	p=0.121	292	116
	Past 6-month SA	1-2 years	Not estimable	—	292	13
		3-4 years	Not estimable	—	292	13
		5+ years	Not estimable	—	292	13
	Lifetime NSSI	1-2 years	0.94 (0.69-1.27)	p=0.691	290	200
		3-4 years	1.21 (0.91-1.60)	p=0.196	290	200
		5+ years	0.96 (0.73-1.26)	p=0.773	290	200
	Past 6-month NSSI	1-2 years	1.70 (0.82-3.53)	p=0.151	290	80
		3-4 years	1.45 (0.66-3.22)	p=0.357	290	80
		5+ years	1.58 (0.80-3.12)	p=0.183	290	80
**Bipolar spectrum**	Lifetime SI	1-2 years	1.19 (0.71-1.98)	p=0.505	79	76
		3-4 years	1.46 (0.94-2.27)	p=0.093	79	76
		5+ years	1.35 (0.91-2.02)	p=0.136	79	76
	Past 6-month SI	1-2 years	0.98 (0.32-3.03)	p=0.972	79	51

Mood disorder subset	Outcome	Diagnostic delay	aRR (95% CI)	p-value	N	Events
		3-4 years	1.11 (0.50-2.49)	p=0.794	79	51
		5+ years	1.02 (0.46-2.22)	p=0.968	79	51
	Lifetime SA	1-2 years	1.19 (0.36-3.89)	p=0.780	79	41
		3-4 years	0.93 (0.35-2.44)	p=0.877	79	41
		5+ years	0.75 (0.28-1.96)	p=0.551	79	41
	Past 6-month SA	1-2 years	Not estimable	—	79	16
		3-4 years	Not estimable	—	79	16
		5+ years	Not estimable	—	79	16
	Lifetime NSSI	1-2 years	1.43 (0.55-3.70)	p=0.462	79	55
		3-4 years	1.44 (0.75-2.76)	p=0.276	79	55
		5+ years	1.20 (0.60-2.41)	p=0.608	79	55
	Past 6-month NSSI	1-2 years	0.65 (0.05-8.01)	p=0.735	78	24
		3-4 years	1.14 (0.08-16.94)	p=0.924	78	24
		5+ years	0.63 (0.05-7.40)	p=0.714	78	24
**MDD only**	Lifetime SI	1-2 years	1.15 (0.91-1.47)	p=0.237	213	186
		3-4 years	1.09 (0.86-1.38)	p=0.484	213	186
		5+ years	0.95 (0.76-1.20)	p=0.665	213	186
	Past 6-month SI	1-2 years	1.26 (0.73-2.16)	p=0.408	210	120
		3-4 years	1.53 (0.94-2.50)	p=0.089	210	120
		5+ years	1.21 (0.76-1.92)	p=0.427	210	120
	Lifetime SA	1-2 years	0.91 (0.51-1.62)	p=0.743	213	88
		3-4 years	1.05 (0.59-1.88)	p=0.867	213	88
		5+ years	0.69 (0.40-1.17)	p=0.170	213	88

Mood disorder subset	Outcome	Diagnostic delay	aRR (95% CI)	p-value	N	Events
	Past 6-month SA	1-2 years	Not estimable	—	213	8
		3-4 years	Not estimable	—	213	8
		5+ years	Not estimable	—	213	8
	Lifetime NSSI	1-2 years	0.85 (0.59-1.22)	p=0.369	211	148
		3-4 years	1.10 (0.78-1.55)	p=0.581	211	148
		5+ years	0.82 (0.60-1.14)	p=0.238	211	148
	Past 6-month NSSI	1-2 years	1.52 (0.63-3.69)	p=0.354	211	56
		3-4 years	1.15 (0.41-3.22)	p=0.791	211	56
		5+ years	1.43 (0.62-3.28)	p=0.397	211	56
**Excluding NOS/other**	Lifetime SI	1-2 years	**1.29 (1.05-1.59)**	**p=0.016**	325	288
		3-4 years	**1.32 (1.09-1.60)**	**p=0.005**	325	288
		5+ years	1.16 (0.95-1.41)	p=0.141	325	288
	Past 6-month SI	1-2 years	1.45 (0.97-2.18)	p=0.073	321	189
		3-4 years	**1.60 (1.10-2.32)**	**p=0.014**	321	189
		5+ years	1.32 (0.92-1.90)	p=0.136	321	189
	Lifetime SA	1-2 years	0.87 (0.56-1.35)	p=0.525	325	136
		3-4 years	1.07 (0.71-1.60)	p=0.751	325	136
		5+ years	0.67 (0.44-1.03)	p=0.066	325	136
	Past 6-month SA	1-2 years	0.71 (0.14-3.73)	p=0.686	325	22
		3-4 years	0.25 (0.04-1.70)	p=0.156	325	22
		5+ years	0.14 (0.02-1.18)	p=0.071	325	22
	Lifetime NSSI	1-2 years	0.94 (0.70-1.26)	p=0.673	323	221
		3-4 years	1.18 (0.90-1.56)	p=0.233	323	221

Mood disorder subset	Outcome	Diagnostic delay	aRR (95% CI)	p-value	N	Events
		5+ years	0.97 (0.74-1.26)	p=0.806	323	221
	Past 6-month NSSI	1-2 years	1.32 (0.67-2.62)	p=0.425	322	85
		3-4 years	1.03 (0.47-2.25)	p=0.943	322	85
		5+ years	1.19 (0.63-2.25)	p=0.590	322	85

Note. aRR = adjusted risk ratio; CI = confidence interval; SI = suicidal ideation; SA = suicide attempt; NSSI = non-suicidal self-injury. Reference category for diagnostic delay is <1 year. Subsets: Unipolar/no bipolar = major depressive and/or persistent depressive disorder without any bipolar diagnosis; Bipolar spectrum = bipolar I, II, or unspecified; MDD only = major depressive disorder with no other mood-disorder type; Excluding NOS/other = excludes mood disorder NOS and 'other'. Models adjust for the same covariates as the main multivariable models (age, race/ethnicity, gender identity, sexual minority status, college degree, employment, US region, urbanicity, self-rated health, mental health comorbidities, adverse childhood experiences, and perceived social support). The bipolar-spectrum subset is small and several estimates are imprecise; interpretations should be made cautiously. Bold = p<0.05. Not estimable = model did not converge to a stable estimate because of the small number of past 6-month suicide attempt events within the subset (quasi-complete separation).

Supplementary Table S2. Diagnostic delay modeled continuously, with a test for departure from linearity.

Outcome	Continuous: aRR per 1-year (95% CI)	Continuous p	Departure from linearity, p	N
Lifetime SI	1.00 (0.99-1.01)	p=0.365	p=0.072	395
Past 6-month SI	1.01 (0.99-1.03)	p=0.269	p=0.328	392
Lifetime SA	**0.96 (0.93-0.99)**	**p=0.010**	**p=0.016**	395
Past 6-month SA	0.93 (0.83-1.05)	p=0.224	p=0.547	395
Lifetime NSSI	1.00 (0.98-1.01)	p=0.721	p=0.621	393
Past 6-month NSSI	1.01 (0.97-1.05)	p=0.677	p=0.709	392

Note. Restricted to participants with valid continuous diagnostic-delay data. The continuous model reports the adjusted risk ratio per 1-year increase. Departure from linearity is a robust Wald test (3 df) of whether a 4-category specification (<1, 1-2, 3-4, 5+ years, derived from the same continuous variable) improves fit over the continuous term. Models adjust for the same covariates as the main multivariable models (age, race/ethnicity, gender identity, sexual minority status, college degree, employment, US region, urbanicity, self-rated health, mental health comorbidities, adverse childhood experiences, and perceived social support). Per-category estimates for the primary survey-reported delay categories appear in Table 2 and Supplementary Table S3. Bold = p<0.05.

Supplementary Table S3. Diagnostic delay and suicidal ideation under alternative specifications and operationalizations.

Outcome	Specification	Level / metric	aRR (95% CI)	p-value	N
**Lifetime SI**	Direct-reported categorical (primary)	1-2 years	**1.23 (1.03-1.47)**	**p=0.021**	413
		3-4 years	**1.26 (1.07-1.49)**	**p=0.006**	413
		5+ years	1.06 (0.90-1.25)	p=0.478	413
	Derived, continuous (per year)	per 1-year increase	1.00 (0.99-1.01)	p=0.365	395
	Derived, granular categories	1-2 years	**1.27 (1.06-1.52)**	**p=0.009**	395
		3-4 years	1.17 (0.97-1.41)	p=0.109	395
		5-9 years	1.15 (0.95-1.38)	p=0.144	395
		10+ years	1.15 (0.96-1.37)	p=0.119	395
**Past 6-month SI**	Direct-reported categorical (primary)	1-2 years	1.25 (0.90-1.72)	p=0.179	409
		3-4 years	**1.37 (1.02-1.85)**	**p=0.038**	409
		5+ years	1.13 (0.85-1.51)	p=0.391	409
	Derived, continuous (per year)	per 1-year increase	1.01 (0.99-1.03)	p=0.269	392
	Derived, granular categories	1-2 years	1.28 (0.92-1.78)	p=0.145	392
		3-4 years	1.36 (0.98-1.89)	p=0.063	392
		5-9 years	1.24 (0.89-1.73)	p=0.206	392
		10+ years	1.25 (0.90-1.74)	p=0.183	392

Note. Each row block is a separate model adjusted for the same covariates as the main multivariable models (age, race/ethnicity, gender identity, sexual minority status, college degree, employment, US region, urbanicity, self-rated health, mental health comorbidities, adverse childhood experiences, and perceived social support). Direct-reported categorical is the single self-reported interval item used in the primary analysis (see Table 2). Derived continuous enters the age-difference measure as years (per 1-year increase). Derived granular categories bin the continuous age-difference measure (<1, 1-2, 3-4, 5-9, 10+ years). Reference category for categorical specifications is <1 year. The reported and derived categorical measures were moderately concordant (67.3% exact agreement; linear-weighted kappa=0.58; N=444). Bold = p<0.05.

Supplementary Figure S1. Restricted cubic spline dose-response between diagnostic delay and suicidal ideation.

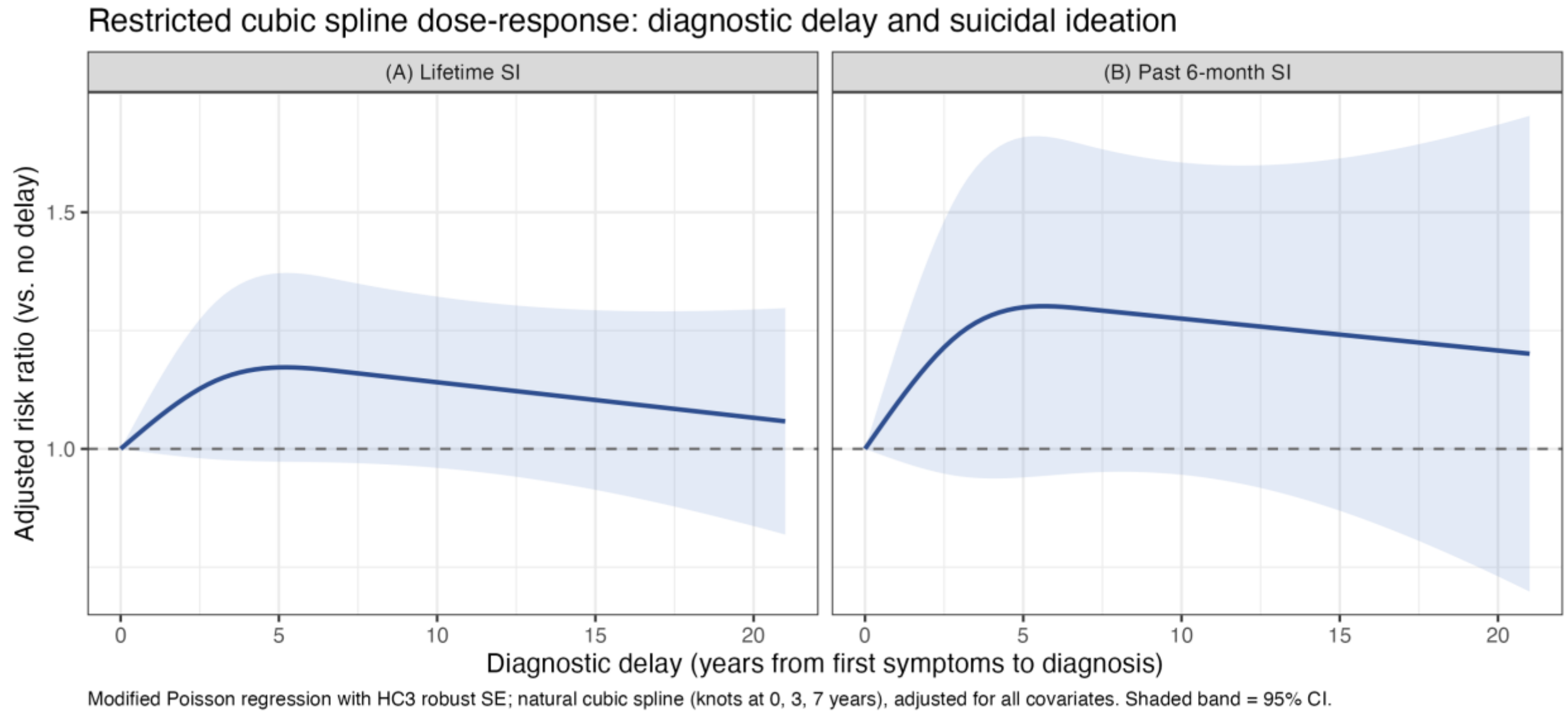

Note. 95% CI = 95% confidence interval. Adjusted risk ratio (relative to no delay) across the observed range of diagnostic delay, from modified Poisson regression with HC3 robust standard errors and a natural cubic spline (knots at 0, 3, 7 years). Models adjust for the same covariates as the main multivariable models (age, race/ethnicity, gender identity, sexual minority status, college degree, employment, US region, urbanicity, self-rated health, mental health comorbidities, adverse childhood experiences, and perceived social support). Analytic sample sizes: Lifetime SI (N=395); Past 6-month SI (N=392). Lifetime SI omnibus spline Wald χ^2 =2.88 (df=2), p=0.237; Past 6-month SI omnibus spline Wald χ^2=2.82 (df=2), p=0.244. Shaded band = 95% CI.

Supplementary Table S4. Parsimonious model for past 6-month suicide attempt (N=455).

Diagnostic delay	aRR (95% CI)	p-value
1-2 years	0.88 (0.39-2.02)	p=0.769
3-4 years	0.68 (0.25-1.89)	p=0.463
5+ years	0.49 (0.19-1.26)	p=0.138

Note. Reduced multivariable model retaining diagnostic delay plus only covariates with a bivariate association at $p<0.05$ (gender identity, urbanicity, any ACEs), given the small number of past 6-month suicide attempts (32 events; N=455). Diagnostic delay remained non-significant, consistent with the main model. Reference category is <1 year. Bold = $p<0.05$.
